# Erythrosine–Iron Oxide Nanoemulsions Combining Photodynamic Therapy with Ferroptosis-Mediated Cytotoxicity in Prostate Cancer

**DOI:** 10.1021/acsomega.6c05709

**Published:** 2026-10-19

**Authors:** Matheus Liberato Ferreira, Giovanna Capaldi Fortunato, Rodolfo Debone Piazza, Marlus Chorilli, Fernando Lucas Primo

**Affiliations:** † Department of Bioprocess and Biotechnology Engineering, School of Pharmaceutical Sciences, São Paulo State University (UNESP), Araraquara 14800-901, SP, Brazil; ‡ School of Pharmaceutical Sciences, São Paulo State University (UNESP), Araraquara 14800-901, SP, Brazil; § Laboratory of Magnetic Materials and Colloids, Department of Physical Chemistry, Institute of Chemistry, São Paulo State University (UNESP), Araraquara 14800-060, SP, Brazil

## Abstract

Prostate cancer remains one of the leading causes of cancer-related death among men worldwide. Photodynamic therapy (PDT) has emerged as a promising strategy to enhance conventional treatments, yet its efficacy is often limited by poor solubility, instability, and a lack of targeting of photosensitizers. Here, we report the development of hybrid erythrosine–iron oxide nanoemulsions (MNE/Ery) produced by vacuum-assisted nanoemulsification. The optimized formulation (0.05 mg·mL^–1^ erythrosine; 0.5 mg·mL^–1^ maghemite) exhibited a hydrodynamic diameter of ∼100 nm, negative surface charge, and strong retention of the photosensitizer’s spectral profile. The iron oxide nanoparticles (20–30 nm) exhibited efficient magnetic heating potential, reaching 42 °C within 5.5 min under an alternating field (418 kHz, 12 kA·m^–1^), indicating suitability for future exploration in magnetic hyperthermia applications. While MNE/Ery maintained high biocompatibility toward healthy cells (HDFn and PNT2), it induced a potent light-triggered cytotoxic response in PC-3 prostate cancer cells. Upon irradiation, the platform delivered a robust, dose-dependent therapeutic effect even at conservative fluences, driven by the combination of maghemite-induced oxidative stress (ferroptosis) and erythrosine-mediated ROS generation. These findings position the MNE/Ery system as a promising nanotechnological platform for PDT with an intrinsic iron-mediated chemotherapeutic effect, with additional potential for future magnetic hyperthermia-based applications.

## Graphical abstract

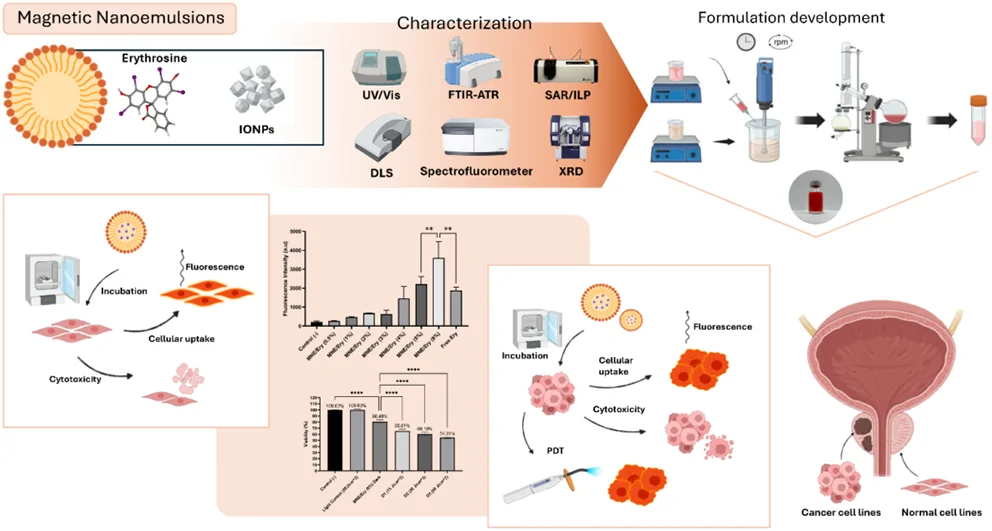

## 1 Introduction

Prostate cancer remains one of the most prevalent malignancies among men and represents a major public health concern because of its high incidence and complex clinical management. According to the International Agency for Research on Cancer (IARC), approximately 1.4 million new cases of prostate cancer and about 375,000 deaths related to the disease were recorded in 2020.^1^ In Brazil, an annual average of 71,730 new cases is estimated for the 2023–2025 triennium, accounting for 30% of all cancers in men, excluding nonmelanoma skin tumors.^2^

The management of prostate cancer remains particularly challenging because of its delicate balance between overtreatment and undertreatment. Overtreatment can expose patients to invasive procedures and significant adverse effects, such as urinary and sexual dysfunction, without a proportional gain in survival, especially in low-grade neoplasia.^3^ Moreover, the complexity involved in the selection and sequencing of therapeutic approaches contributes to the heterogeneity of clinical outcomes.^4^ These challenges underscore the urgent need for more selective, patient-centered, and minimally invasive strategies that maintain oncological efficacy while preserving quality of life.^5,6^

Within this context, photodynamic therapy (PDT) has emerged as a promising alternative for prostate cancer management. This minimally invasive technique is capable of inducing localized tumor ablation through the activation of photosensitizing agents by light irradiation, leading to reactive oxygen species (ROS) generation and subsequent cell death.^7,8^ However, clinical translation remains limited due to the poor solubility of many photosensitizers, their instability in biological environments, and consequently reduced bioavailability, which collectively compromise therapeutic efficacy.^9^

To overcome these physicochemical and pharmacokinetic limitations, nanoemulsion-based delivery systems have gained an increasing amount of attention. These nanostructured platforms improve the solubility and stability of photosensitizers, improve their bioavailability, and reduce systemic cytotoxicity, thereby providing a more efficient and controlled therapeutic delivery.^10,11^ In parallel, the integration of photodynamic therapy (PDT) with iron oxide nanoparticles (IONPs) has been demonstrated to increase cellular damage by combining photodynamical ROS generation with intrinsic dysfunctional iron metabolism in cancer cells, resulting in enhanced tumor cell ablation.^12,13^ This therapeutic combination can be further optimized through nanoemulsion-based systems, enhancing overall treatment efficacy.^14,15^

Erythrosine, a xanthene-based photosensitizer, exhibits strong absorption in the visible region, presenting high singlet oxygen quantum yield and low cytotoxicity, making it an established agent for PDT.^16,17^ Although erythrosine has been investigated in various oncological applications, its potential in prostate cancer therapy remains unexplored.^18,19^ Complementarily, iron oxide nanoparticles (IONPs) have been widely explored for cancer therapy due to their biocompatibility, superparamagnetic behavior, and ability to generate localized heating under alternating magnetic fields, enabling MHT.^20–22^

Based on the scenario of hybrid nanostructured systems, this study proposes the evaluation of the stability, cytotoxicity, and efficacy of a magnetic nanoemulsion (MNE/Ery) containing iron oxide nanoparticles and a xanthene photosensitizer, erythrosine. This investigative strategy aimed to evaluate the possible effects of combining maghemite nanoparticles with erythrosine in photodynamic therapy in prostate cancer cells.

## 2 Materials and Methods

### 2.1 Magnetic Nanoemulsion Formulation Method

Magnetic nanoemulsions (MNEs) were prepared by the nanoemulsification via a vacuum-assisted method (Figure 1). The aqueous phase, containing Pluronic F68 and iron oxide nanoparticles (IONPs), was initially homogenized using an Ultra-Turrax T25 (IKA, Germany) at low rotational speed to disperse the components while minimizing shear-induced loss of the superparamagnetic nanoparticles. The organic phase (Lipoid S100 and Erythrosine dissolved in acetone) was then added dropwise onto the aqueous phase, with the Ultra-Turrax speed concomitantly increased to the optimized high-shear homogenization condition. Solvent removal was subsequently performed under reduced pressure at 55 °C using a rotary evaporator Hei-VAP Expert Control (Heidolph, Germany).

**Figure 1:**
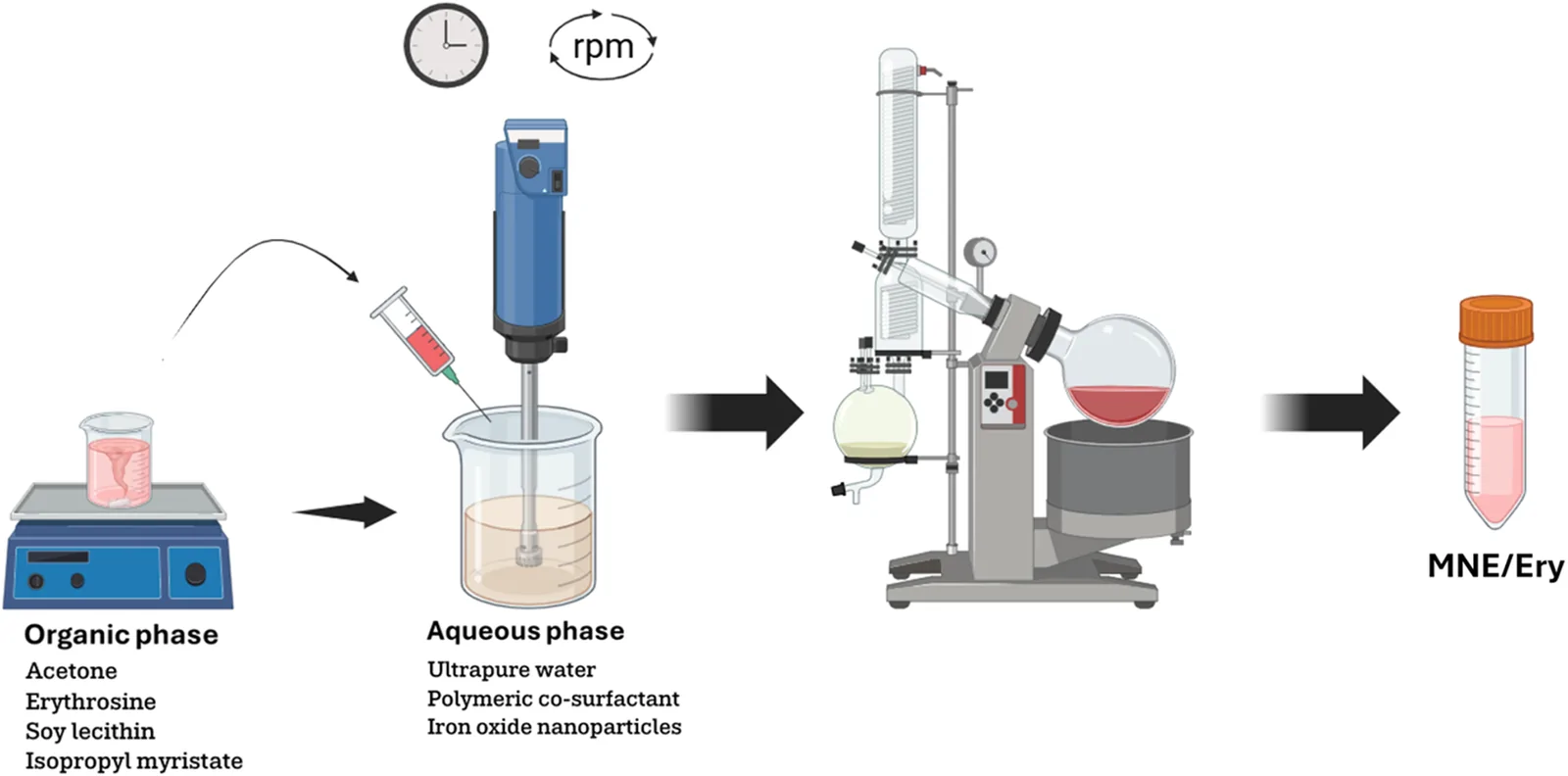
Preparation of magnetic nanoemulsions via vacuum-assisted emulsification. Schematic illustration of the methodology integrating Ultra-Turrax homogenization and reduced-pressure solvent evaporation (rotary evaporation) for nanostructure formation.

Formulations containing 1.0 mg·mL^–1^ of iron oxide nanoparticles (IONPs) were prepared under low (6,000 rpm) and high (12,000 rpm) homogenization speeds, and three homogenization durations: short (5 min), medium (10 min), and long (15 min). Subsequently, the combination of parameters that yielded the best results was used to develop magnetic nanoemulsions containing 0.05 mg·mL^–1^ of Erythrosine (MNE/Ery) and three different IONP concentrations: 1.0, 0.5, and 0.1 mg·mL^–1^.

### 2.2 Determination of Hydrodynamic Diameter and Polydispersity Index by Dynamic Light Scattering and Zeta Potential by Electrophoretic Mobility

The measurements were performed using a Zetasizer Nano ZS (Malvern Instruments, UK) to determine the hydrodynamic diameter (HD), zeta potential (ζ), and polydispersity index (PdI). For the analysis of magnetic nanoemulsions (MNEs), samples were diluted 1:100 in ultrapure water. In the case of iron oxide nanoparticles (IONPs), stabilization was achieved by citrate adsorption following the procedure described by Yang et al. (2014).^23^

### 2.3 Fourier Transform Attenuated Reflectance Infrared Spectroscopy (FTIR-ATR) Analysis

The chemical composition of MNE/Ery and all formulation components was analyzed by FTIR-ATR using a Bruker α II spectrometer at room temperature. Spectra were recorded over a range of 400–4000 cm^–1^ with a spectral resolution of 4 cm^–1^ and analyzed in terms of percent transmittance.

### 2.4 IONP Magnetic Characterization: SAR and ILP

The heating efficiency of IONPs was evaluated by measuring temperature change over time in a suspension containing 12.5 mg of IONPs in 1 mL of distilled water using the DM100 Series instrument (NanoScale Biomagnetics, Spain). Measurements were performed under alternating magnetic fields of 12 kA·m^–1^ at frequencies of 202 and 418 kHz.

The specific absorption rate (SAR) was calculated according to eq 1, where the initial temperature was set as ambient temperature, is the specific heat capacity of water (4.18 J·g^–1^·°C^–1^), *m*_Liq_ is the mass of water, *m*_IONP_ is the mass of nanoparticles, and Δ*T*/Δ*t* represents the rate of temperature change over time:

1 $$SAR=\frac{\left(c_{Liq}\times m_{Liq}\right)}{m_{MNP}}\times \frac{\Delta T}{\Delta t}$$

The intrinsic loss power (ILP) was calculated by normalizing SAR to the field amplitude and frequency (eq 2), allowing comparison independent of the applied magnetic field parameters:

2 $$\mathrm{ILP}=\frac{\mathrm{SAR}}{{f\times H}^{2}}$$

where *f* is the applied frequency and *H* is the field amplitude.

### 2.5 X-ray Diffraction

XRD analyses were performed using a Smartlab SE diffractometer (Rigaku, Tokyo, Japan) at the Central Laboratory for Chemical and Structural Analyses (LACAQUE), UNESP. Measurements employed Cu Kα radiation (λ = 1.54186 Å) in reflection geometry (θ/2θ), with a 2θ range of 20–80°, step size of 0.02°, and scan speed of 5°/min. Soller slits (2.5°), incident slit (0.5°), divergence slit (10 mm), and open receiver slit were used. Average crystallite sizes were estimated using the Debye–Scherrer equation:^24^


3 $${\left\langle D\right\rangle }_{\left(311\right)}=\frac{K\times \lambda }{\mathrm{FWHM}\times \cos\theta }$$

where *K* = 0.9, λ is the X-ray wavelength, θ is the Bragg angle, and FWHM is the full width at half-maximum of the (311) peak.

### 2.6 2D and 3D UV–Vis and Fluorescence Spectroscopy

Absorption spectra of Erythrosine in MNEs were acquired using a Genesys 10S UV–vis spectrophotometer (Thermo Fisher Scientific, USA) over 400–800 nm with 1 cm quartz cuvettes. Fluorescence emission spectra were recorded using an RF-6000 spectrofluorimeter (Shimadzu, Japan) over 300–800 nm. Two-dimensional fluorescence measurements used 526 nm excitation with 5 nm slit widths and a scanning speed of 600 nm·min^–1^. Three-dimensional spectra were obtained from 430–595 nm excitation and 520–740 nm emission. Samples were diluted 5-fold to a final concentration of 5 μmol·L^–1^. Free Erythrosine solutions were prepared at the same concentration.

### 2.7 Development of an Analytical Method for Erythrosine Quantification

The analytical method for erythrosine quantification was validated regarding linearity, precision, accuracy, and sensitivity limits (LOD and LOQ), following standard validation guidelines. The calibration curve was constructed in acetonitrile within the concentration range of 5–25 μmol L^–1^ (*R* ^2^ > 0.99). Precision (repeatability and intermediate) and accuracy were evaluated at three concentration levels (5, 12.5, and 25 μmol L^–1^) and expressed as Relative Standard Deviation (RSD) and percent recovery, respectively. Limits of detection (LOD) and quantification (LOQ) were calculated based on the standard deviation of the *y*-intercept and the slope of the regression line.

### 2.8 Atomic Force Microscopy

Surface topography of the nanoemulsions was characterized by atomic force microscopy (AFM), a technique that enables three-dimensional imaging at the nanoscale resolution. Samples of MNE/Ery were deposited as a droplet onto freshly cleaved mica substrates and allowed to dry under ambient conditions prior to analysis. Imaging was carried out in tapping mode using a Scanning Probe Microscope SPM-9600 (Shimadzu, Kyoto, Japan), operated through the manufacturer’s SPM Online software. A silicon cantilever measuring 124 μm in length was employed, with resonance frequencies within the 324–369 kHz range and spring constants between 34 and 51 N·m^–1^.

### 2.9 Analytical Method for IONP Quantification

The quantification of iron oxide nanoparticles (IONPs) was performed according to the method described by Wu et al. (2012),^25^ based on the formation of an iron–thiocyanate complex. Briefly, iron oxide nanoparticles were digested in 2 mol·L^–1^ HCl and subsequently reacted with potassium thiocyanate. The absorbance of the resulting complex was measured at 480 nm using a Genesys 10S UV–Vis spectrophotometer (Thermo Fischer Scientific, USA). Calibration was established using FeCl_3_ standard solutions within the 0.0025–0.080 mg mL^–1^ range.

### 2.10 Cellular Culture

Human neonatal dermal fibroblasts (HDFn, PCS-201-010),^26^ normal prostate epithelial cells (PNT2, BCRJ-0366), and prostate adenocarcinoma cell lines (PC3, CRL-1435) were obtained from the American Type Culture Collection (ATCC) and the Rio de Janeiro Cell Bank (BCRJ). Cells were cultured in Dulbecco’s Modified Eagle Medium (DMEM, Gibco, USA) supplemented with 10% fetal bovine serum (FBS, Vitrocell, Brazil) and 1% penicillin–streptomycin (10,000 U/mL). All cell lines were maintained at 37 °C in a humidified atmosphere containing 5% CO_2_.

#### 2.10.1 MNE/Ery Cellular Uptake Assays

Cellular uptake was quantified on the basis of erythrosine fluorescence. Cells were seeded and treated under conditions identical to the cytotoxicity assay. After 6 and 24 h of incubation (and 48h for PNT2 cells), monolayers were washed with phosphate-buffered saline (PBS) to remove noninternalized formulation. The fluorescence intensity was recorded using a PerkinElmer EnSpire MLD 2300 microplate reader (PerkinElmer, USA) at an excitation wavelength of 530 nm.

#### 2.10.2 Nanoformulation Cytotoxicity Assays

In vitro cytotoxicity was evaluated according to ISO 10993-5 guidelines. Cells were seeded in 96-well plates and exposed to MNE/Ery (0.5–50%), blank nanoemulsion (MNE-VZ), free erythrosine solution, and magnetic nanoparticle suspension (*n* = 3). Dimethyl sulfoxide (DMSO) served as the positive control. Cell viability was assessed using the Resazurin reduction assay. After 6 and 24 h of exposure (and up to 48 h for PNT2 cells), cells were washed with PBS and incubated with resazurin solution for 4 h. Fluorescence intensity was quantified at excitation and emission wavelengths of 560 and 590 nm, respectively.

### 2.11 Photodynamic Efficacy Assay

Photodynamic efficacy was assessed using a concentration range of 0.5–8% (v/v) with incubation times of 6 and 24 h. In all sets, monolayers were washed and immersed in PBS prior to irradiation. An LED device (Optilight Max, 1200 mW, 500 nm) was used to deliver fluences of 15, 30, and 50 J·cm^–2^. Postirradiation, cells were incubated in fresh medium for 24 h before viability quantification via the Resazurin mitochondrial metabolism assay.

## 3 Results and Discussion

### 3.1 Evaluation of Physical-Chemical Analysis of Iron Oxide Nanoparticles

#### 3.1.1 Size, Zeta potential, and PdI

Iron oxide nanoparticles (IONPs) exhibit a strong tendency to aggregate due to their low solubility in aqueous media and the magnetic dipole–dipole interactions between particles. This behavior is highly dependent on the characteristics of the dispersing medium, which influence both the aggregation kinetics and sedimentation rate. Factors such as ionic strength, pH, and the presence of stabilizing agents play a crucial role in determining the colloidal stability of IONPs.^27–29^

In this context, dynamic light scattering (DLS) analyses provided relevant information regarding the physicochemical parameters and size distribution profiles of the magnetic nanoemulsions (MNEs) under study.^30^ Measurements were performed in aqueous medium without surface treatment and after citrate modification of the magnetic nanoparticles (MNPs), as summarized in Table 1.

**Table 1:** Parameters Obtained by DLS of Size, Zeta Potential, and PdI of IONPs before and after Citrate Coating in Aqueous Media

Parameters	IONP	IONP-Citrate
Hydrodynamic diameter (d.nm)	417.8	30.6
Zeta Potential (mV)	–4.4	–30.7
Polydispersity index	0.51	0.38

Bare iron oxide nanoparticles (IONPs) typically exhibit a near-neutral ζ-potential due to the balance of surface hydroxyl groups. Upon functionalization with citric acid, the surface charge density changes significantly, resulting in a distinctly negative ζ-potential. This shift is driven by the strong coordination of at least one carboxylate group of citric acid to the iron surface ions, while the remaining uncoordinated carboxyl groups orient toward the aqueous medium and undergo deprotonation.^23^ Consequently, this enhanced electrostatic repulsion accounts for the observed decrease in both the hydrodynamic diameter and the polydispersity index (PDI), confirming the successful surface modification and improved colloidal dispersion stability.

#### 3.1.2 X-ray Diffraction (XRD)

X-ray diffraction analysis provided clear evidence for the crystalline phase of the iron oxide nanoparticles (Figure 2). The diffractogram exhibits the characteristic reflections of a cubic spinel structure, with peaks located at 30.24, 35.72, 43.41, 53.84, 57.41, 63.06, and 74.64° (2θ), corresponding to the (220), (311), (400), (422), (511), (440), and (533) planes, respectively.^31^ These values closely match the reference pattern for maghemite reported by the ICDD and show a closer alignment with maghemite than with magnetite.^32^

**Figure 2:**
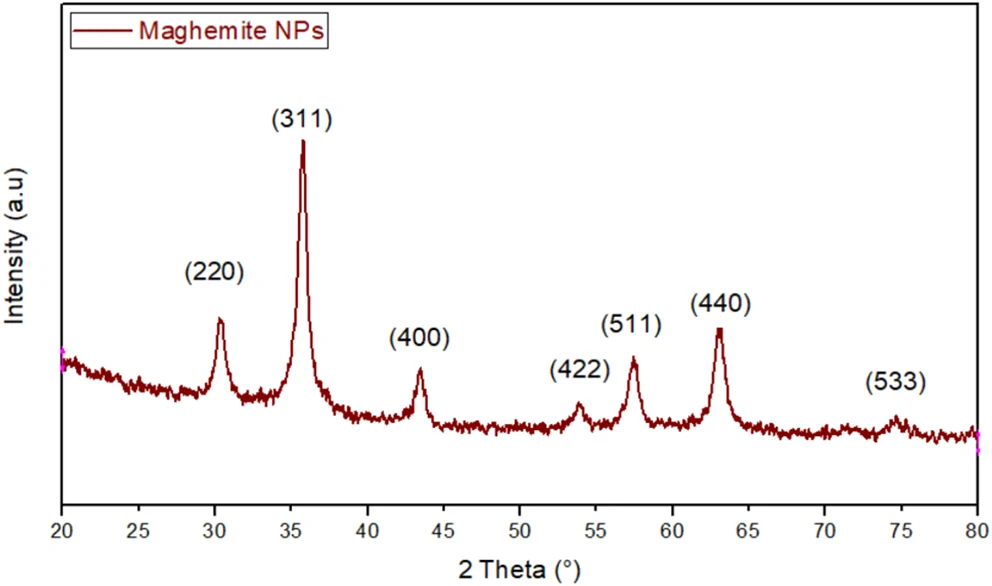
X-ray diffraction (XRD) pattern of the iron oxide nanoparticles. The characteristic peaks correspond to the (220), (311), (400), (422), (511), and (440) crystallographic planes, consistent with the cubic spinel structure of maghemite.

Using the most intense and best-resolved reflection (311), the crystallite size estimated via the Debye–Scherrer equation was approximately 22.3 nm. This value is consistent with the hydrodynamic size distribution obtained from DLS, indicating the presence of well-defined nanocrystallites and supporting the assignment of the sample to the γ-Fe_2_O_3_ phase.^33^

#### 3.1.3 Evaluation of Specific Absorption Rate (SAR) and Intrinsic Loss Power (ILP)

The magnetothermal response of IONPs was evaluated over a 9 min exposure period, as shown in Figure 3. At a concentration of 12.5 mg mL^–1^, application of an alternating magnetic field with *H* = 12 kA m^–1^ and *f* = 418 kHz resulted in a rapid temperature rise, reaching 42 °C within 5 min 28 s, corresponding to a SAR of 42.0 W g^–1^. In contrast, when the frequency was reduced to 202 kHz while maintaining the same field amplitude, the suspension reached only 31.4 °C after the full 9 min exposure. The calculated intrinsic loss power (ILP) was 0.70 nH m^2^ kg^–1^, which aligns with the range reported by Kallumadil et al. (2009)^34^ for commercial iron oxide nanoparticles (0.15–3.12 nH m^2^ kg^–1^). This value is also consistent with Piazza et al. (2020),^35^ who reported SAR and ILP values of 32.8 W g^–1^ and 0.39 nH m^2^ kg^–1^, respectively, for uncoated maghemite. Under these conditions (418 kHz; 12 kA m^–1^), the IONPs demonstrated effective magnetic heating within the biological safety limit proposed by Atkinson-Brezovich (H × *f* < 5 × 10^9^ A·m^–1^·s^–1^), indicating their suitability for localized biomedical hyperthermia applications.^36–38^

**Figure 3:**
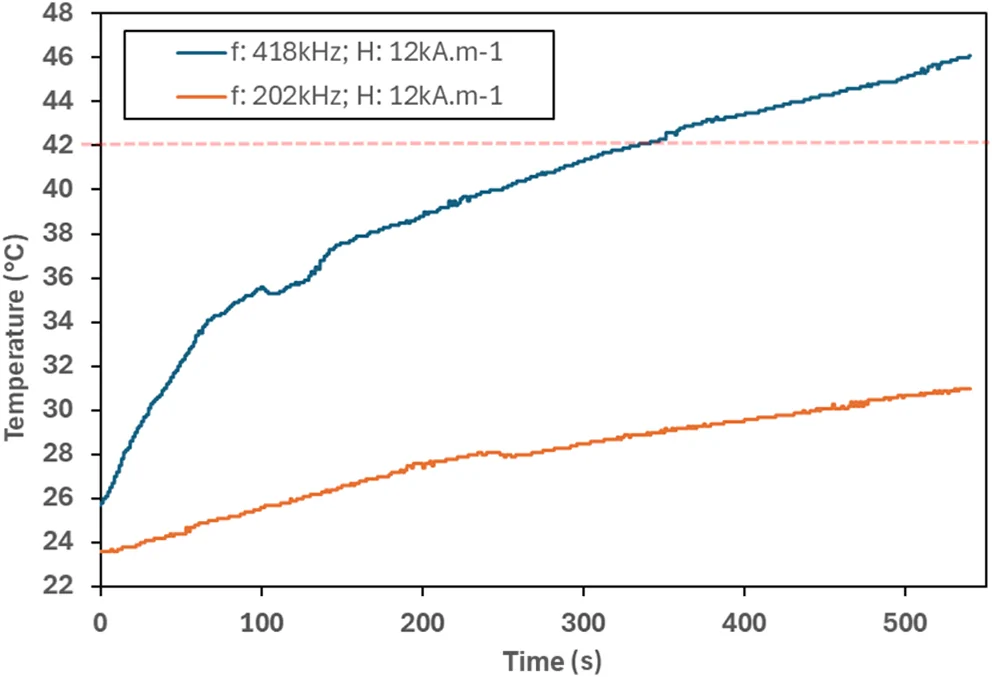
Calorimetric heating profiles of iron oxide nanoparticles under alternating magnetic fields (AMF). Time-dependent temperature evolution at fixed field strength (*H* = 12 kA.m^–1^) and varying frequencies (*f* = 202 and 428 kHz). The slope of the curves indicates the frequency-dependent Specific Absorption Rate (SAR).

### 3.2 Magnetic Nanoemulsions (MNE) Characterization

#### 3.2.1 Influence of Synthesis Parameters on MNE’s Size, Zeta Potential, and PdI

Nine formulations were evaluated for hydrodynamic diameter, Zeta potential, and PdI across different combinations of homogenization speed and time. Among these tested conditions, 12,000 rpm for 10 min yielded the most favorable visual and statistical outcomes. Hydrodynamic diameter showed no significant differences among formulations. Zeta potential remained close to −30 mV overall, with slightly more negative values observed at 12,000 rpm/10 min, indicating satisfactory electrostatic stabilization. PdI values presented a statistically significant difference between 12,000 rpm/10 min and 6,000 rpm/10 min. Nonetheless, a general decrease in PdI was observed with longer homogenization times, particularly at 12,000 rpm.

Visual inspection after 90 days corroborated these findings: samples processed at 6000 rpm exhibited phase separation, whereas those prepared at 12,000 rpm remained homogeneous with a stable brown coloration. Overall, higher homogenization speeds improved the nanoparticle dispersion and prevented phase separation.

#### 3.2.2 Time Stability of MNE/Ery

DLS analyses showed that the hydrodynamic diameter (DH) of the formulations (1.0, 0.5, and 0.1 mg·mL^–1^) remained around 100 nm over time, with no statistically significant differences between measurement dates (Figure 4). The polydispersity index (PDI) remained approximately 0.4 for all samples and showed no significant variation. The PDI, an indicator of system uniformity based on the particle size distribution, indicates that lower values correspond to greater homogeneity and, consequently, higher stability.^39^

**Figure 4:**
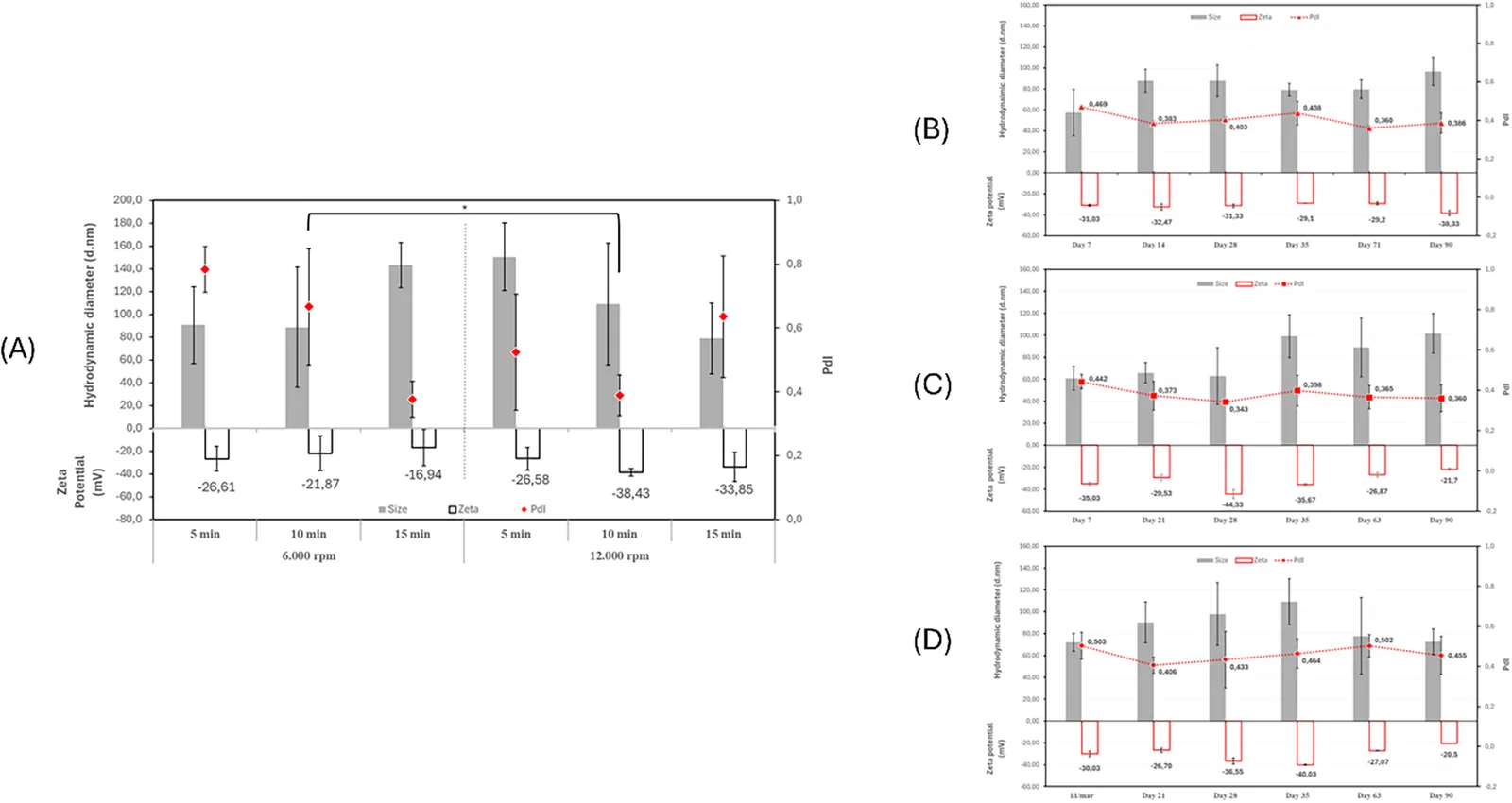
Optimization of processing parameters and long-term colloidal stability of magnetic nanoemulsions. (A) Effect of homogenization speed (6,000 and 12,000 rpm) and duration (5, 10, and 15 min) on the initial droplet size, PZ, and PDI. (B–D) Evaluation of physical stability over 90 days for MNE/Ery formulations at different IONP concentrations (0.1, 0.5, and 1.0 mg mL^–1^), monitoring the same previous parameters using DLS.

Zeta potential (ZP), which measures the effective surface charge of particles via electrophoretic mobility, exhibited significant fluctuations over time. Nanoparticles with ZP values between +10 and −10 mV are generally less stable, whereas those with higher absolute values (>±30 mV) display increased stability due to electrostatic repulsion.^40,41^ Despite these ZP variations, the maintenance of DH and PDI suggests that the overall stability of the MNE/Ery nanoemulsions was not compromised; however, the observed fluctuations in surface charge highlight the need for further investigation to elucidate their origin and potential implications for long-term colloidal behavior.

#### 3.2.3 Photophysical Properties of the MNE/Ery Formulation

##### 3.2.3.1 2D Absorption and Fluorescence Analysis

This assay was conducted to evaluate whether erythrosine preserves its characteristic absorption and fluorescence behavior upon incorporation into the MNE/erythrosine system, in comparison with the free photosensitizer dissolved in acetonitrile. In addition, a two-dimensional fluorescence emission profile of the erythrosine-loaded nanoemulsion was obtained as a complementary quality control parameter, providing a reliable means to monitor formulation consistency and stability over time.^42^

Despite the well-established solvatochromism of erythrosine, it is characterized by absorption and emission maxima at 530 and 550 nm in aqueous media and red-shifted bands at 546 and 565 nm in acetonitrile.^43,44^ The fluorescence emission profile of erythrosine associated with the nanoemulsion closely overlaps with that of the free dye under comparable conditions (Figure 5A,B).

**Figure 5:**
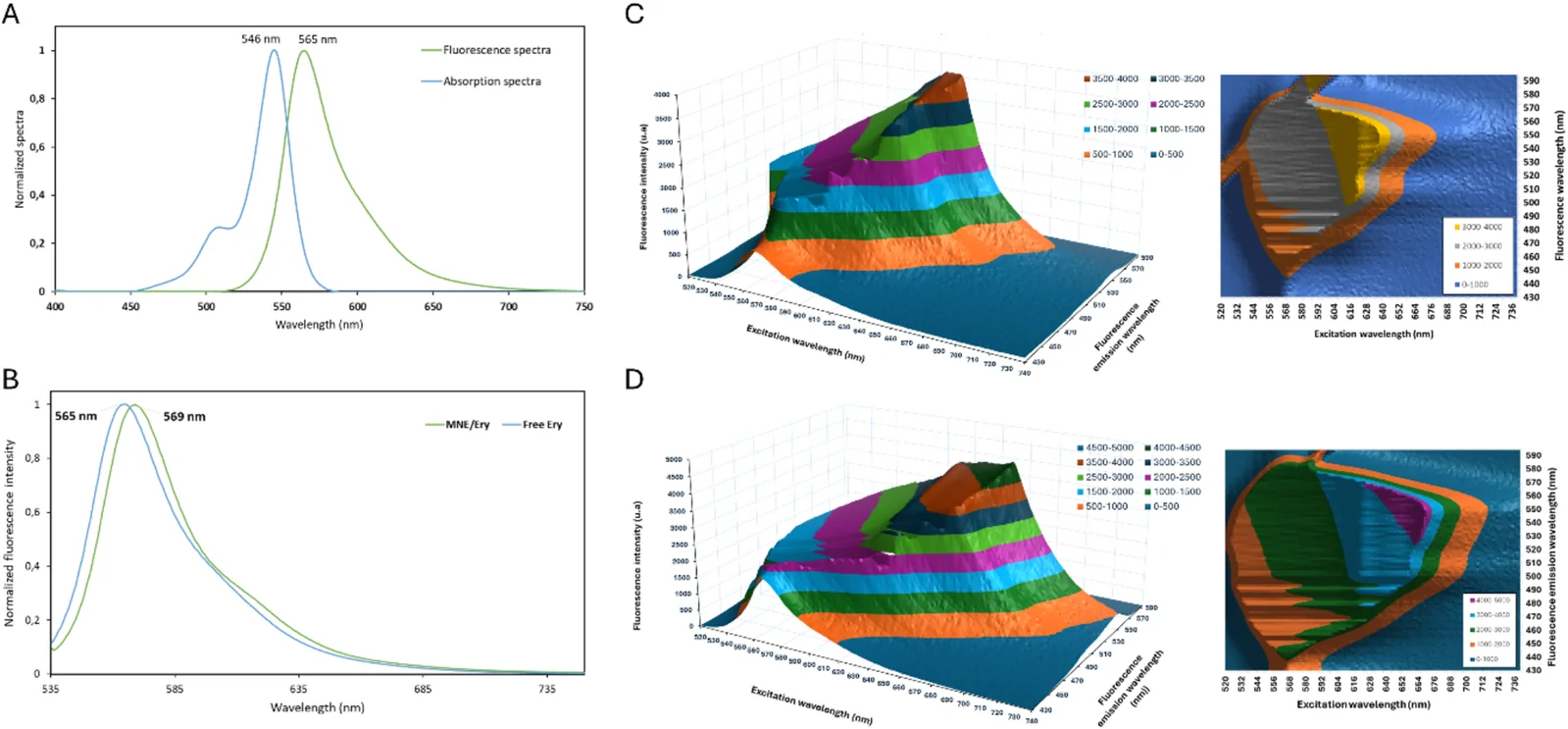
Photophysical characterization and spectral integrity of erythrosine within the magnetic nanoemulsion. (A) Normalized UV–vis absorption and fluorescence spectra of erythrosine. (B) Comparative fluorescence emission spectra of free and nanoemulsified erythrosine (λ_EX_ = 546 nm), highlighting the preservation of the Erythrosine’s emissive profile. (C, D) Representative 3D Excitation–Emission Matrix (EEM) fluorescence maps for (C) free erythrosine and (D) erythrosine-loaded nanoemulsions, featuring top-view surface projections to illustrate intensity distribution and spectral landscape. Measurements were performed within the excitation range of 520–740 nm and emission range of 430–594 nm, using a constant spectral slit width of 5 nm for both excitation and emission.

Such solvent-induced shifts are well documented and arise from differential stabilization of the ground and excited states by the surrounding medium, including solvent reorganization and specific solute–solvent interactions.^45^ Importantly, the preservation of the spectral position and shape upon nanoemulsification indicates that incorporation into the MNE does not significantly perturb the electronic structure of erythrosine, a behavior consistent with reports for other photosensitizers formulated in nanostructured systems.^46^

##### 3.2.3.2 3D Fluorescence Spectra Analysis

The three-dimensional absorption and emission profiles were evaluated by using surface plots and corresponding contour maps to facilitate direct comparison between samples. The analysis encompassed emission wavelengths from 520 to 740 nm and excitation wavelengths of 430 and 595 nm. Upon association of the photosensitizer with the nanostructured system, a noticeable broadening of the fluorescence emission band was observed, manifested as an increased base width of the emission peak relative to that of free erythrosine.

This effect was accompanied by minor variations in the emission wavelength range and a subtle shift of the emission maximum (Figure 5A,B). Such changes are attributed to light scattering due to the presence of formulation constituents in the medium rather than to intrinsic electronic perturbations of the photosensitizer.

Consistent with the two-dimensional spectral analyses, the three-dimensional emission surface of nanoemulsified erythrosine exhibits a high degree of correspondence to that of the free photosensitizer. This spectral similarity indicates that nanoemulsification does not significantly alter the photophysical properties of erythrosine, supporting the suitability of the formulation for applications in the PDT.

#### 3.2.4 Infrared spectroscopy (ATR-FTIR)

ATR-FTIR spectroscopy was employed to assess the chemical interactions and structural integrity of the Erythrosine-loaded (MNE/Ery) and blank (MNE-VZ) nanoemulsions relative to their individual components (Figure 6). Consistent with literature and ChemicalBook (2025)^46^ data, a broad band around 3360 cm^–1^, attributed to residual water O–H stretching, was observed in all spectra.^47^ The band at ∼1640 cm^–1^ corresponds to the water bending mode and 2400–2300 cm^–1^ of atmospheric CO_2_, a phenomenon frequently reported in nanoparticle samples.^48,49^ Bands at 2920–2850 cm^–1^, present in isopropyl myristate, Lipoid S100, and Pluronic F68, reflect the C–H stretching of aliphatic chains, while the sharp, intense peak at 1740–1720 cm^–1^ confirms the presence of ester carbonyl groups (C=O) from the lipids. Furthermore, peaks between 1340 and 1100 cm^–1^ evidence C–O and C–O–C stretching characteristic of alcohols, ethers, and polyols.^50–52^

**Figure 6:**
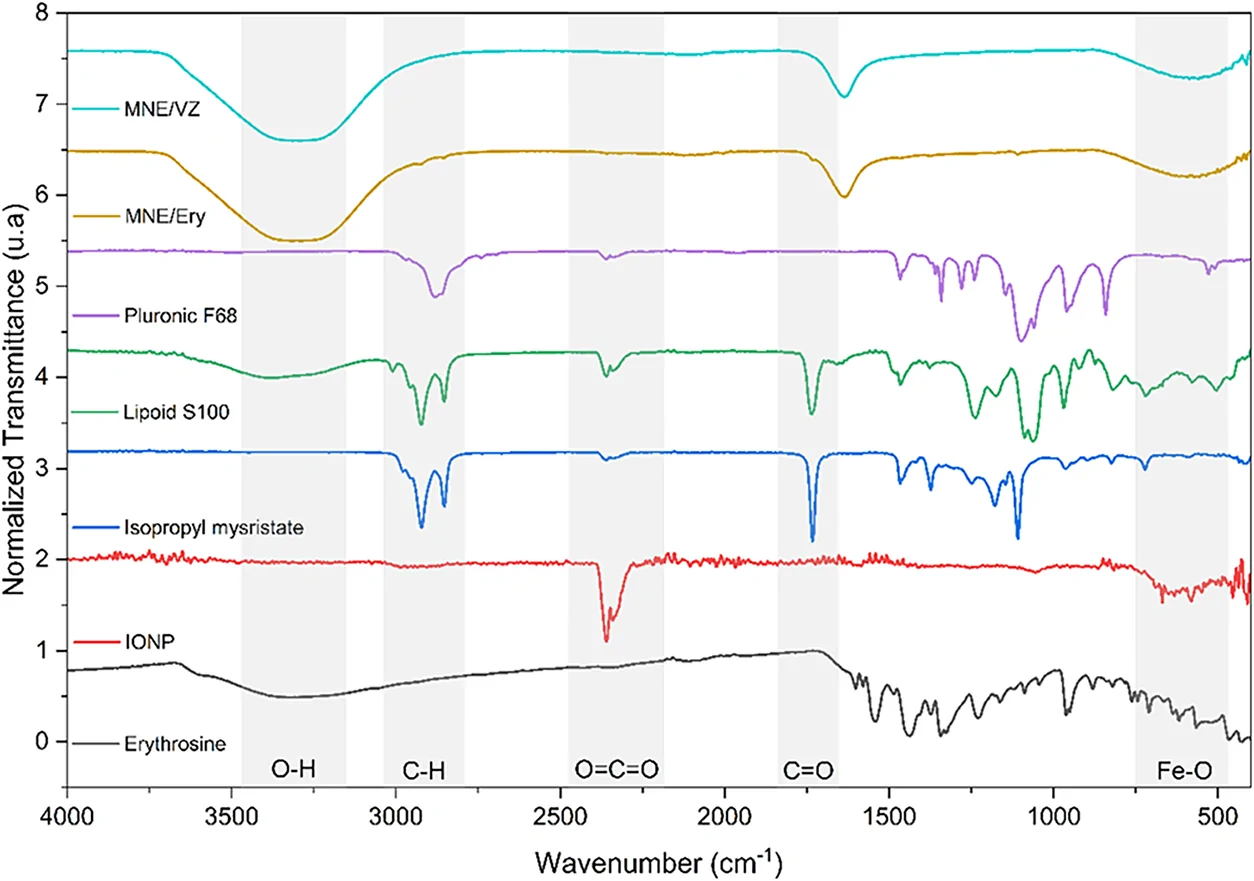
FTIR spectra of pure formulation components, blank nanoemulsions (MNE/VZ), and erythrosine-loaded nanoemulsions (MNE/Ery).

FTIR analysis of the formulation revealed spectra predominantly dominated by the intense vibrational bands of water, which effectively suppressed the signals of the organic molecules present in minor proportions. While bands in the 900–500 cm^–1^ region could theoretically overlap with photosensitizer signals, their presence in the blank formulation (MNE-VZ) confirms they arise from Fe–O lattice vibrations, explicitly identifying the maghemite core. Thus, unlike the encapsulated drug, which is shielded by the lipid matrix, the inorganic magnetic core retains a detectable spectroscopic signature.^53^

#### 3.2.5 Erythrosine Quantification

The analytical method demonstrated linearity (*R* ^2^ > 0.99) within the range of 5–25 μmol L^–1^. Precision and accuracy parameters were within acceptable limits (RSD < 5%), and the LOD and LOQ were determined as 1.12 and 3.40 μmol L^–1^, respectively. Applying this method, the total erythrosine content in the formulation was quantified following the destabilization of the nanoemulsion with acetonitrile. The analysis revealed a high erythrosine recovery of 77.2 ± 0.5%, corresponding to a final concentration of 0.0386 mg·mL^–1^ in the MNE/Ery formulation. The low standard deviation confirms the high reproducibility of the synthesis method.

#### 3.2.6 IONP Quantification

Magnetic nanoparticle (MNP) content was quantified spectrophotometrically following acid digestion. The analysis revealed a final concentration of 0.419 ± 0.019 mg·mL^–1^, representing an incorporation efficiency of approximately 84% relative to the theoretical input (0.5 mg·mL^–1^). Although this indicates good retention of the theoretical concentration, the slight reduction may be attributed to minor losses of iron oxide nanoparticles adhering to the Ultra-Turrax shaft during homogenization.

#### 3.2.7 Atomic Force Microscopy

Three-dimensional AFM topographic analysis of MNE/Ery (Figure 7) revealed droplets with predominantly spherical morphology, consistent with the size variability observed by dynamic light scattering (DLS). Given the heterogeneous nature of the sample, comprising nanoemulsion droplets alongside iron oxide nanoparticles and their aggregates, particle sizes were evaluated individually rather than as a single-averaged population. From the topographic profiles (*n* = 47 particles analyzed), droplet sizes of 106.34, 99.37, 56.58, and 37.66 nm were measured, indicating a mixed population of nanoemulsion droplets around 100 nm, together with smaller structures compatible with iron oxide nanoparticle aggregates and isolated iron oxide nanoparticles. This size distribution is in agreement with the particle size range obtained by an X-ray diffraction (XRD) analysis.

**Figure 7:**
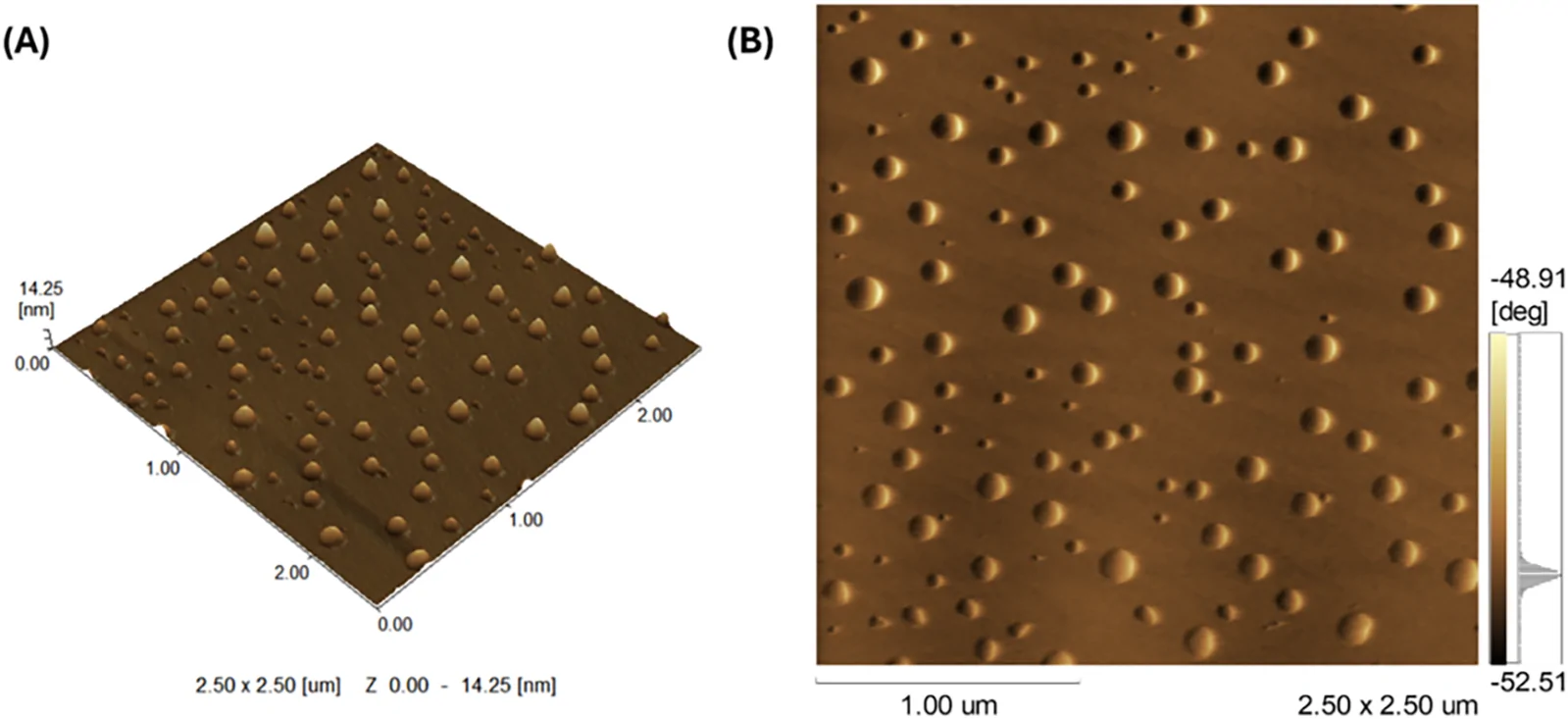
Two- and three-dimensional photomicrography of the topographic morphology profile of MNE/Ery (A and B).

### 3.3 *In Vitro* Biological Evaluation

#### 3.3.1 Cytotoxicity and Erythrosine Cellular Uptake Assessment

##### 3.3.1.1 Human Dermal Fibroblast Neonatal (HDFn)

According to ISO 10993-5, the cytotoxic effect is defined by a reduction in the cell viability of more than 30%. Thus, the cell viability analysis performed with resazurin after 6 h of contact with the formulations showed no cytotoxicity associated with MNE/Ery. Even after 24 h, the formulation remained noncytotoxic, except for the highest concentration (MNE/Ery 50%). However, the suspension containing only IONPs (free IONPs) and free photosensitizer solution (free Ery) showed a reduction of more than 30% in cell viability. This behavior indicates lower toxicity of nanoemulsified systems (MNE/Ery) compared to the components in free form (Figure 8A).

**Figure 8:**
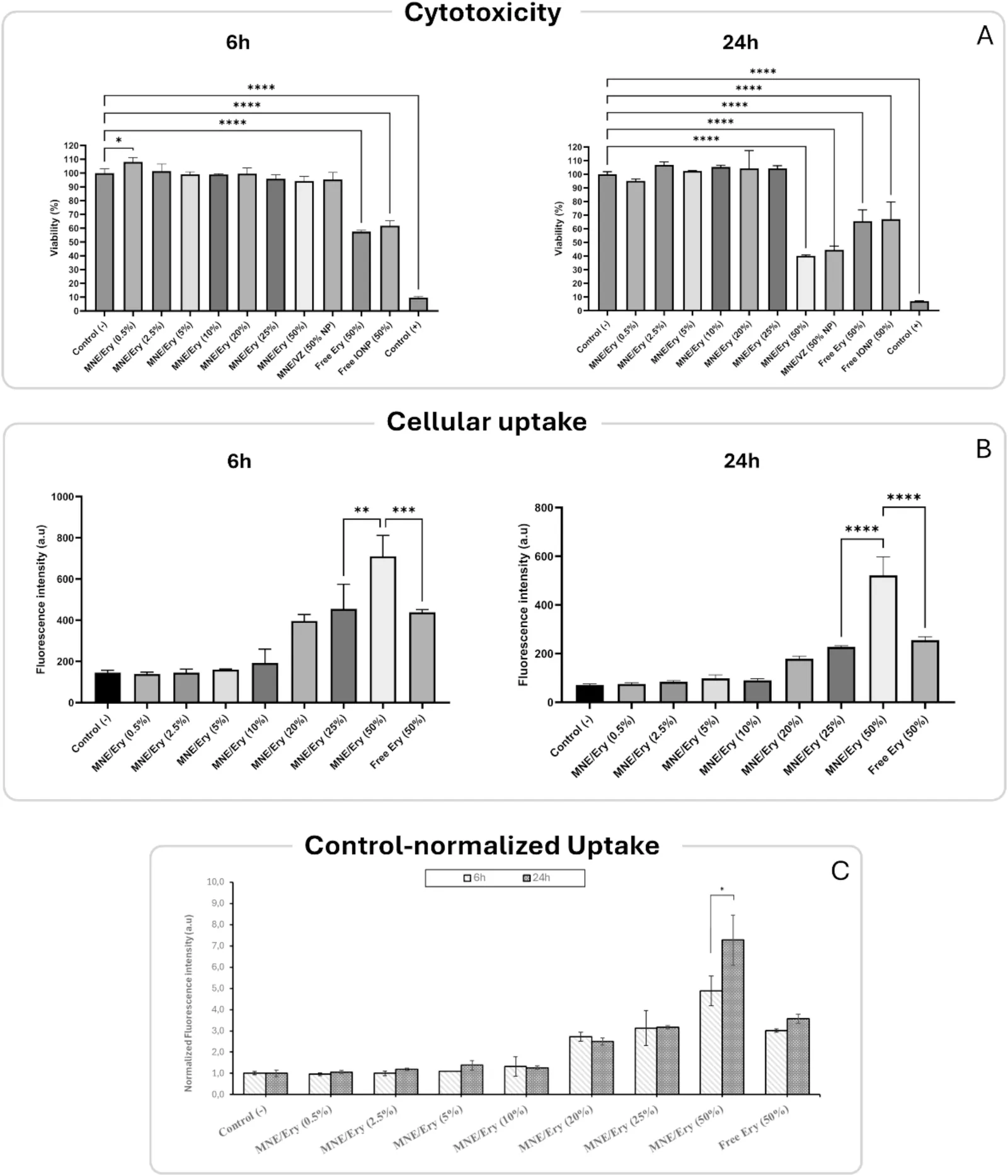
*In vitro* cytotoxicity and cellular uptake of MNE/Ery in HDFn cells. (A) Cell viability was evaluated at 6 and 24 h of incubation with varying concentrations of the formulation (0.5–50% v/v), free erythrosine (50%), free NPs (50%), and a negative control. (B) Cellular uptake profile measured by erythrosine fluorescence intensity (λ_EX_ = 530 nm) across the same concentration range for 6 and 24 h. (C) Normalized cellular uptake comparing the 6 and 24 h incubation periods to highlight internalization based on the control. Statistical significance was determined by One-way ANOVA followed by Dunnett and Tukey’s post-hoc test (*p* < 0.05).

For cellular uptake, the fluorescence intensity of erythrosine exhibited a positive correlation with increasing formulation concentrations in the medium (Figure 8B). To ensure data accuracy, fluorescence intensity was normalized to the respective controls, revealing a significant signal increase after 24 h compared with 6 h of exposure (Figure 8C). Notably, the MNE formulation displayed significantly higher fluorescence intensity compared to free erythrosine at equivalent concentrations (*p* < 0.05). This distinct behavior underscores the superior internalization efficiency of MNE/Ery, suggesting that the nanocarrier facilitates cellular uptake while cytotoxic effects remain restricted to the highest doses.

##### 3.3.1.2 Prostate Normal Tissue (PNT2)

The cytotoxicity assays performed on PNT2 cells demonstrated a favorable biocompatibility profile after 6 and 24 h of exposure, with signs of toxicity observed only at the highest concentration at the 24 h time point (Figure 9A). Furthermore, free erythrosine and suspended nanoparticles generally maintained cell viability above 75%, although the IONP suspension dropped below this threshold after 24 h. Finally, at 48 h, significant cytotoxicity was observed starting from the 20% MNE/Ery concentration, with even more pronounced toxic effects observed for the free components (<60%).

**Figure 9:**
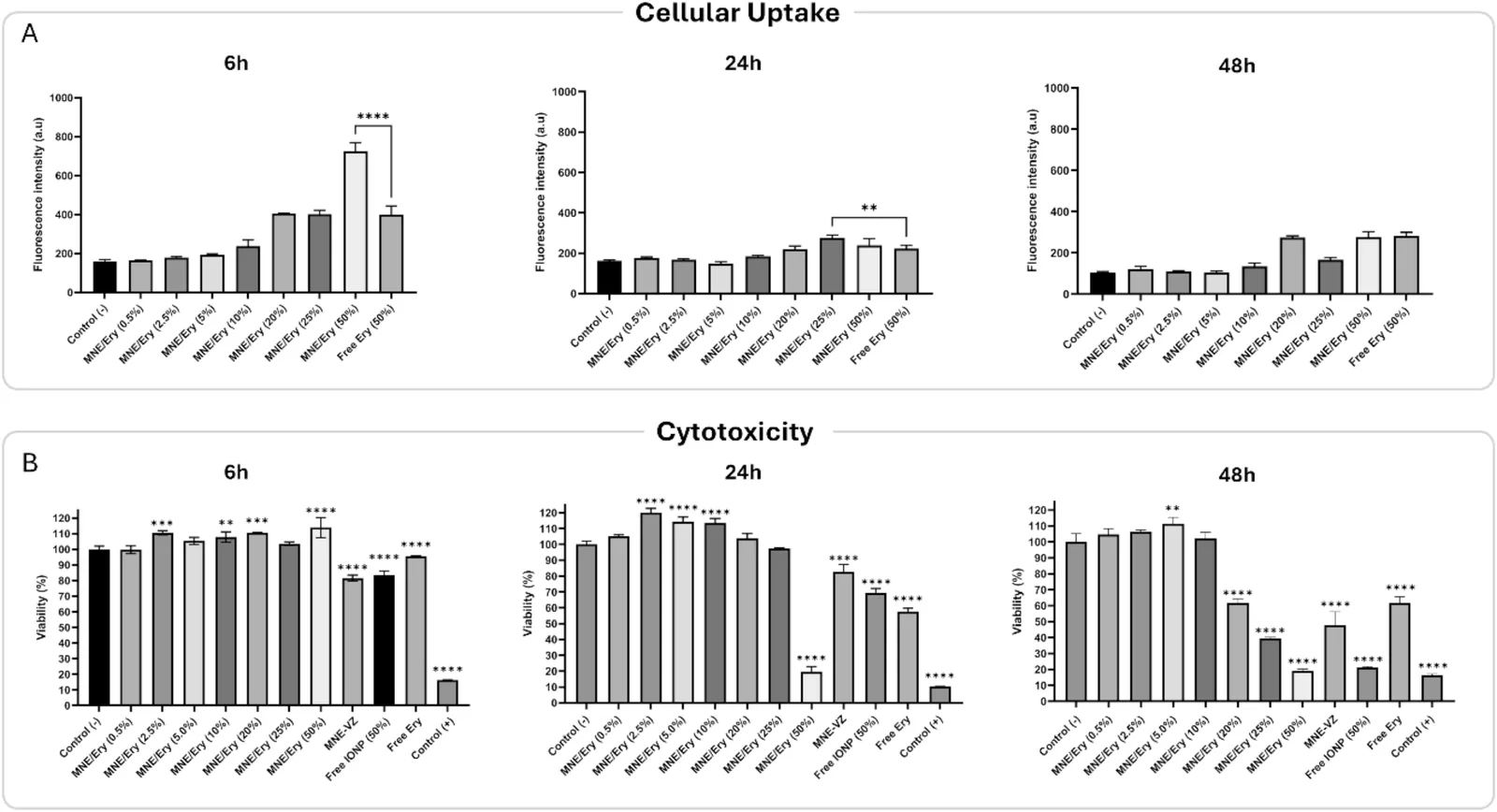
*In vitro* cytotoxicity and cellular uptake of MNE/Ery in PNT2 cells. (A) Cellular uptake profile measured by erythrosine fluorescence intensity (λ_EX_ = 530 nm) across the same concentration range for 6, 24, and 48 h. (B) Cell viability was evaluated at 6, 24, and 48 h of incubation with varying concentrations of the formulation (0.5–50% v/v), free erythrosine (50%), free NPs (50%), and a negative control. Statistical significance was determined by One-way ANOVA followed by Dunnett and Tukey’s post-hoc test (*p* < 0.05).

The cellular uptake profile revealed a distinct trend with regard to the erythrosine fluorescence signal. While the maximum fluorescence intensity was recorded after 6 h of exposure across most concentrations, a significant signal reduction was noted at 24 and 48 h (Figure 9B). As described by Adjei et al. (2014),^54^ nanoparticles internalized via endocytic pathways are typically routed to lysosomes, where the payload is subjected to hydrolytic enzymes and an acidic environment. Crucially, spectroscopic studies indicate that erythrosine absorbance is pH-dependent, reaching maximum values above pH 6 but undergoing spectral shifts and reduced absorbance in acidic media.^55^ Therefore, the peak intensity at 6 h likely reflects the accumulation of the PS in early endosomes, whereas the subsequent signal loss at 24 h suggests that the observed fluorescence decrease could be associated with protonation-induced quenching within lysosomes in addition to potential enzymatic degradation.

##### 3.3.1.3 Prostatic Adenocarcinoma (PC-3)

Initial assessments revealed that higher concentrations of the MNE/Ery formulation (10–50% v/v) exhibited pronounced intrinsic dark cytotoxicity against tumor cell lines within a 6 h incubation period. To accurately isolate the photodynamic efficacy, ensuring that cell death is driven by light irradiation rather than the inherent toxicity of the nanomaterial, it was necessary to establish a noncytotoxic baseline. Consequently, lower concentrations ranging from 0.5 to 8.0% (v/v) were selected for further evaluation (Figure 10B). Crucially, at these reduced concentrations, no biologically significant cytotoxic effects were observed in the dark after 6 and 24 h of incubation. Although minor, statistically significant fluctuations in mitochondrial metabolic activity were detected among the groups, cell viability consistently remained above the 80% threshold across all tested conditions. These results demonstrate the biocompatibility of the MNE/Ery system within this specific concentration range, validating its suitability for subsequent photodynamic assays.

**Figure 10:**
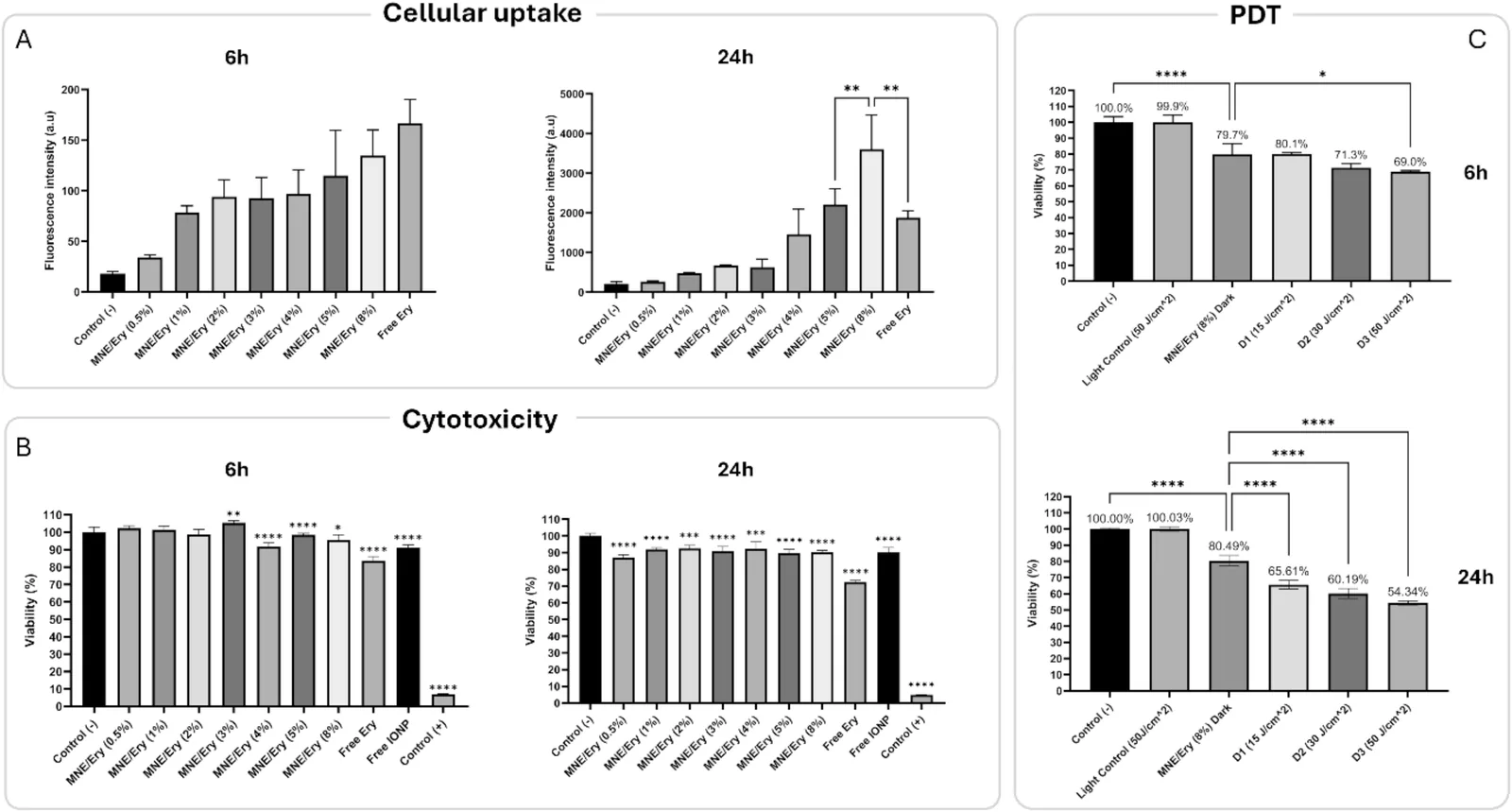
*In vitro* cytotoxicity and cellular uptake of MNE/Ery in PC3 cells. (A) Cell viability was evaluated at 6 and 24 h of incubation with varying concentrations of the formulation (0.5–8% v/v), free erythrosine (8%), free NPs (8%), and a negative control. (B) Cellular uptake profile measured by erythrosine fluorescence intensity (λ_EX_ = 530 nm) across the same concentration range for 6 and 24 h. (C) PDT assays using negative control, light control, dark control, and three different light doses (15, 30, 50 J·cm^–2^). Statistical significance was determined by One-way ANOVA followed by Dunnett and Tukey’s post-hoc test (*p* < 0.05).

The cellular uptake kinetics in the PC3 tumor cell line revealed a distinct internalization profile (Figure 10B). Following a 6 h incubation period, the intracellular erythrosine signal remained minimal at lower concentrations. Notably, at this time point, no statistically significant difference in cellular uptake was observed between the MNE/Ery formulation and free erythrosine, even at the higher fractions of 5 and 8% (v/v). However, extending the incubation time to 24 h resulted in a progressive concentration-dependent increase in the intracellular fluorescence signal. Crucially, at 24 h, the MNE/Ery nanocarrier demonstrated a statistically significant enhancement in cellular uptake compared to free erythrosine at the maximum tested concentration of 8% (v/v) as well as at the 5% (v/v) fraction.

These findings indicate that the internalization of the MNE/Ery system by PC3 cells is a complex, time-dependent process. The delayed uptake observed initially suggests that lower formulation concentrations might alter the internalization regime. Nevertheless, following a prolonged 24 h exposure, the nanocarrier achieves substantial and significantly superior intracellular accumulation relative to that of the free photosensitizer (Figure 10A). Furthermore, this enhanced intracellular retention highlights a well-established advantage of nanoscale delivery systems in protecting the molecule from recognition by cellular efflux pumps, thereby helping to overcome inherent resistance mechanisms and bypass the rapid expulsion of the active molecules.^56^

#### 3.3.2 MNE/Ery Photodynamic Evaluation

##### 3.3.2.1 Prostatic Adenocarcinoma (PC3)

The photodynamic performance of MNE/Ery against PC-3 revealed an interplay between incubation kinetics and light dose response (Figure 10C). At 6 h of incubation, a significant difference was observed only at the highest light dose (50 J cm^–2^) compared to the dark control (*p* < 0.05), aligning with the lower internalization rates previously observed for this shorter interval. In contrast, the 24 h treatment revealed the most interesting result through robust light-dose-dependent cytotoxicity, where all irradiated groups showed significant differences relative to the dark control. This gradual reduction in viability across increasing light fluences demonstrates that the cellular preconditioning, induced by basal dark stress, renders PC-3 cells highly vulnerable to the subsequent oxidative burst generated during irradiation.

To further support this selective dark cytotoxicity, the differential iron metabolism in tumor cell lines emerges as a key mechanism. Neoplastic cells typically exhibit dysregulated iron homeostasis, characterized by an expanded intracellular labile iron pool and impaired export routes.^57^ Once internalized, the magnetic nanoparticles within the formulation can undergo degradation in the acidic lysosomal environment, releasing ferric and ferrous ions.^58^ In tumor cells, this iron surplus acts as a potent catalyst for redox reactions, exacerbating basal oxidative stress. This phenomenon promotes the localized peroxidation of glycerophospholipids, potentially inducing structural mitochondrial damage and triggering ferroptosis, an iron-dependent programmed cell death pathway.^59^ Consequently, the formulation’s core composition intrinsically acts as an agent of metabolic instability, corroborating the cellular preconditioning effect prior to photodynamic therapy.

## 4 Conclusions

In summary, this study reported the development and physicochemical characterization of an erythrosine-loaded magnetic nanoemulsion (MNE/Ery). The nanoformulation exhibited favorable colloidal stability while preserving the photophysical activity of the photosensitizer. In vitro biological evaluations demonstrated the biocompatibility of the nanocarrier in healthy cell lines while suggesting a potentially targeted antitumor activity against prostate adenocarcinoma cells (PC-3). This differential susceptibility might be attributed to basal metabolic stress, possibly driven by lysosomal iron release from the maghemite NPs and subsequent activation of ferroptosis-related pathways. Rather than a limitation, this cellular preconditioning could potentially act synergistically with photodynamic therapy (PDT), possibly enabling enhanced cell death under clinically viable light doses. Ultimately, the MNE/Ery platform presents itself as a promising nanomedicine strategy, offering preliminary evidence as a combined focal treatment integrating PDT with an intrinsic iron-mediated chemotherapeutic effect (ferroptosis) against aggressive prostate cancer.
